# EASIX, Modified EASIX and Simplified EASIX as an Early Predictor for Intensive Care Unit Admission and Mortality in Severe COVID-19 Patients

**DOI:** 10.3390/jpm12071022

**Published:** 2022-06-21

**Authors:** Aleksander Zińczuk, Marta Rorat, Krzysztof Simon, Tomasz Jurek

**Affiliations:** 1 Department of Forensic Medicine, Wroclaw Medical University, 50-367 Wrocław, Poland; marta.rorat@umw.edu.pl (M.R.); tomasz.jurek@umw.edu.pl (T.J.); 2 1st Department of Infectious Diseases, J. Gromkowski Specialist Regional Hospital, 51-149 Wrocław, Poland; krzysztof.simon@umw.edu.pl; 3 Department of Infectious Diseases and Hepatology, Wroclaw Medical University, 50-367 Wrocław, Poland

**Keywords:** Endothelial Activation and Stress Index, SARS-CoV-2, endotheliitis, inflammation

## Abstract

COVID-19 receives a lot of attention due to its threat to global public health. Research is ongoing to find universal methods to assess the baseline health status of a patient to determine prognosis and management strategies. This study aims to assess the predictive potential of the EASIX (Endothelial Activation and Stress Index) and two of its modifications (mEASIX and sEASIX) in terms of the need for admission to the ICU (intensive care unit), the use of IMV (invasive mechanical ventilation) and death due to COVID-19. The medical data of 370 severely ill patients hospitalised in the COVID-19 departments of the Regional Specialist Hospital in Wroclaw (Poland), including the ICU, were analysed retrospectively. The mortality rate in the group studied was 65.7% (243 cases). In the case of all three indices, EASIX, mEASIX and sEASIX, there was a statistically significant correlation between the need for admission to the ICU (*p* = 0.026, *p* = 0.019, *p* = 0.001, respectively) and the risk of death (*p* < 0.001). In terms of the risk of death, the high values of the assessed indices (EASIX ≥ 2.36, mEASIX ≥ 704.03, sEASIX ≥ 3.81) were characterised by low sensitivity (≤40%), high specificity (approximately 90%) and low NPV (negative predictive value) (approximately 40%) with high PPV (positive predictive value) (approximately 80%). Due to the ease of implementation and the low cost of performing basic laboratory tests, the above-mentioned indices can be used as an additional, but not universal tool for the initial assessment of the health condition of patients admitted to the hospital.

## 1. Introduction

COVID-19 (coronavirus disease 2019) is a multi-organ disorder that primarily affects the respiratory system [1,2]. The disease, caused by SARS-CoV-2, is associated with high mortality among hospitalised patients. The very first Chinese epidemiological data from the beginning of the pandemic indicated severe SARS-CoV-2 infection in the case of approximately 16% of patients, of whom 3.2% were critically ill patients who developed thromboembolic disorders, sepsis/septic shock, multi-organ failure and death [3,4]. A meta-analysis conducted by Jie Li et al. that involved more than 280,000 patients (based on data up to 6 April 2020), showed that severe disease occurred in 22.9% of cases and mortality reached 5.6% [5]. Current epidemiological data shows that the global COVID-19 mortality rate does not exceed 2% (approximately 1.64%) [6].

Knowledge of risk factors and the ability to predict the severity of an infection is constantly evolving. Data from numerous studies proves the existence of a number of risk factors for a severe course of the disease and death, such as: older age, male gender, smoking, pregnancy, use of immunosuppressants, or comorbidities including chronic heart, lung, liver and kidney diseases, diabetes, obesity and malignancies [7,8,9,10,11]. The correlation between the severity of the patient’s condition and the results of routinely performed laboratory tests (C-reactive protein, lymphocytes, neutrophils, urea, troponin I, bilirubin, ferritin, D-dimer, creatinine kinase and lactate dehydrogenase), or the intensification of inflammatory changes in imaging tests was also demonstrated [4,5,12,13,14]. However, research is ongoing to find tools that would be universal and easy to use, in the event of a large number of infections, for early assessment of the risk of deterioration of a patient’s condition based on baseline parameters.

The pathophysiology of a severe course of COVID-19 is associated with, among other things, damage to the vascular endothelium, uncontrolled release of cytokines and pro-inflammatory factors, and abnormalities in the composition and functioning of immune system cells, which ultimately lead to multiple organ failure [15,16]. By binding to endothelial cells via ACE2 receptors [17], SARS-CoV-2 activates a signalling cascade, which leads to the release of pro-inflammatory cytokines that trigger the coagulation process. Consequently, a condition known as thromboinflammation develops, which is the basis of thromboembolic complications that make up the CAC (COVID-19 associated coagulopathy) [18]. Thus, endothelial dysfunction appears to be the key pathophysiological factor of a severe course and complications due to COVID-19, as confirmed by early analyses, performed at the beginning of the pandemic, of laboratory markers in patients infected with SARS-CoV-2 and the presence of high levels of pro-inflammatory cytokines that are endothelial stress markers (angiopoietin-2 and CXCL8) [19,20,21,22,23,24].

The above mentioned discovery justified an attempt to use the EASIX index (Endothelial Activation and Stress Index), which is the correlation between popular laboratory markers of endothelial dysfunction: lactate dehydrogenase (LDH) activity, creatinine concentration and thrombocytes count, for the baseline assessment of the condition of patients. Initially, the EASIX index was described by Luft et al. as a predictor of death in patients with acute graft versus host disease (GvHD) after allogeneic haematopoietic marrow cell transplantation it correlated well with the concentration of endothelial stress markers, particularly with CXCL-8/IL-18 and free IL-18 [25,26]. EASIX was also found to be a strong predictor of the severity of COVID-19 and mortality due to the disease. As in the case of haematological diseases, it shows a significant correlation with the concentration of endothelial and inflammatory markers (ANG2, sTM, ST2, CXCL8, CXCL9, IL-18) [27]. To date, there are only three publications on such an assessment and in each of them statistically significant results were achieved justifying further research in that direction [27,28,29].

The aim of this study is to assess the possibility of using the conventional EASIX index and its two modifications, the simplified EASIX (sEASIX) and the modified EASIX (mEASIX), as predictors of the need for admission to the ICU (intensive care unit), the use of IMV (invasive mechanical ventilation) and death among patients with COVID-19 in a severe clinical condition. To our knowledge, this is the first publication on the use of modified indices (mEASIX, sEASIX) to assess the condition of such patients.

In terms of the management of healthcare systems with limited access to intensive care units, which also applies to Poland, the identification of patients who are at risk of severe illness, in particular those at risk of death from COVID-19, is of key importance. Providing such patients with appropriate specialist monitoring will increase their chances of survival thanks to timely and appropriately targeted treatment. The use of a simple tool, such as the EASIX index, in combination with other popular diagnostic methods to assess the risk of an unfavourable course of the disease is a good opportunity to achieve the above-mentioned goal. The hypothesis of the study is that high values of EASIX and the modified sEASIX and mEASIX coefficients are correlated with the undesirable clinical outcome (death, ICU admission and invasive mechanical ventilation).

## 2. Materials and Methods

Retrospective analysis of the medical data of patients with severe COVID-19 hospitalised in the period from 6 March 2020 to 30 June 2021 in a specialist hospital dedicated to the treatment of patients with COVID-19 in Lower Silesia (Poland). The study covered 370 consecutively admitted patients (out of a total of 2407 patients hospitalised during that time) with SARS-CoV-2 infection confirmed by a real-time RT-PCR (reverse transcription-polymerase chain reaction) or rapid antigen test (PanBio COVID-19 Ag Rapid Test Device by Abbott), who developed severe (WHO 3 and 4) disease at different times of hospitalisation, regardless of their condition upon admission, so they could be admitted with WHO 1 or 2 at baseline [30]. Patients with a mild to moderate (WHO 1 and 2) course of the disease, those who were transferred to other hospitals and their survival/non-survival status unknown or those who died before the first basic laboratory tests on admission to the hospital were performed, were excluded from the study.

Demographics (age and gender), presence of comorbidities, the time from onset of symptoms to the time of admission to the hospital, baseline blood saturation measured with the use of room-air pulse oximetry, course of hospitalisation (length, day of admission to the ICU, duration of ICU stay) were taken into account in the study. With regard to the laboratory tests, the values of the parameters on the 1st day of hospitalisation were considered: C-reactive protein (CRP), procalcitonin (PCT) and ferritin concentration, lactate dehydrogenase (LDH) activity, white blood count (WBC), neutrophils count (NEU), lymphocytes count (LYM), haemoglobin (HGB), platelet count (PLT), D-Dimer and creatinine concentration, international normalised ratio (INR), aspartate aminotransferase (AST) and alanine aminotransferase (ALT) activity. The baseline condition of the patient was assessed using the 4-point WHO scale (1-mild disease, symptomatic COVID-19 patients without evidence of viral pneumonia or hypoxia; 2-moderate disease, clinical signs of pneumonia (fever, cough, dyspnoea, fast breathing), no signs of severe pneumonia, SpO_2_ ≥ 90% on room-air; 3-severe disease, clinical signs of pneumonia plus one of the following: respiratory rate > 30/min, severe respiratory distress, SpO_2_ < 90% on room-air; 4-critical disease, ARDS, sepsis or septic shock) [30]. The study also considered the method and duration of individual oxygen therapy (oxygen therapy using a mask with a reservoir at a minimum flow of 10 L/min, HFNO-high flow nasal oxygenation, NIV-non-invasive ventilation, IMV-invasive mechanical ventilation). In the absence of specific recommendations on the application of different types of oxygen therapy in the period assessed, qualification for treatment was performed individually by the attending physician based on the results of blood saturation measurements taken using a pulse oximeter, blood gas tests, imaging studies and general medical condition. The decision to intubate and perform invasive mechanical ventilation was made by an anaesthesiology and ICU specialist. Disease progression was defined as meeting the severe case criteria according to the WHO classification (≥3). If the status of the admitted patient was considered to be 3 according to WHO, progression was assessed as the moment the method of oxygen therapy was changed-the need for escalation. Overall, 369 out of 370 patients had laboratory tests performed on admission, which were necessary for the calculation of: EASIX according to the standard formula by Luft et al. (LDH [U/L] × Creatinine [mg/dL]/platelet count [G/L]) and modified sEASIX (LDH [U/L]/platelet count [G/L]) and mEASIX (LDH [U/L] × CRP [mg/dL]/platelet count [G/L]) according to Pennisi et al. [26,31]. In this study, a different unit of CRP was used, which is commonly used in Poland and worldwide-(mg/L) was used instead of (mg/dL) and the mEASIX index was assessed without conversion to that unit.

The study was conducted according to the guidelines of the Declaration of Helsinki, and approved by the Bioethics Committee of Wroclaw Medical University (Decision no. KB-826/2020, dated 17 December 2020). The requirement to obtain informed consent from the patients to use their medical data was waived by the bioethics committee. To perform the retrospective study, data was anonymised before being entered into the database and analysed.

## 3. Statistical Analysis

Descriptive statistics were presented: for quantitative variables as mean values with SD (standard deviation) or/and median with IQR (interquartile range), for qualitative variables as absolute count with percentage. Quantitative variables (including EASIX, mEASIX, sEASIX) were transformed and analysed using log_2._ For this, Spearman’s rank–order correlation was used. Qualitative variables were compared using the Mann–Whitney test and tau–Kendall correlation. Optimal log_2_-EASIX, log_2_-mEASIX and log_2_-sEASIX cut-off values for death were determined by the Youden Index.

The T-test with Cox-adjustment was used for univariable analysis. The optimal cut-off point for quantitative variables was calculated using the Youden Index. Receiver operating characteristics (ROC) curves analysis was performed to assess the effectiveness of said points (sensitivity, specificity, area under the ROC curve (AUC)).

Analysis was performed using Statistica (version 13.3, TIBCO Software Inc., Palo Alto, CA, USA) for Windows. All statistical tests were considered significant with the *p*-value < 0.05.

## 4. Results

The characteristics of the study group divided into subgroups in terms of clinical outcome are presented in Table 1. Overall, 243 patients out of 370 (65.7%) died. The main cause of death among patients was respiratory failure (86.4%), and other disorders predominated in the remaining cases (13.6%), but respiratory failure accompanied them (septic shock in the course of secondary bacterial infection, progression of neoplastic disease, decompensated liver cirrhosis, ischaemic or haemorrhagic stroke, subarachnoid haemorrhage, post-surgery multi-organ failure, sudden circulatory arrest, haemorrhagic stroke and acute renal failure).

Patients who died were older (*p* < 0.001, cut-off ≥ 72 years), more frequently burdened with circulatory system diseases (*p* = 0.009), malignant neoplasms (*p* = 0.046), and chronic renal failure (*p* = 0.001). They were also characterised by higher LDH activity (*p* = 0.026), elevated levels of D-dimer (*p* = 0.018) and creatinine (*p* = 0.016) at the time of admission. They required more frequent application of NIV (*p* < 0.001) and IMV (*p* < 0.001), and less frequent application of HFNO (*p* < 0.001) for a shorter period. Furthermore, the disease progression was faster in the group of non-survivors—those patients required earlier admission to the hospital (calculated from the onset of the symptoms) compared to patients from the group of survivors (*p* = 0.026).

Parameters (quantitative variables) determined on admission to the hospital, showing a statistically significant correlation in the context of prediction of mortality, were analysed in terms of the ability to explain the risk of death. None of the parameters achieved AUC > 0.7—the data is presented in Table 2.

**Figure 1:**
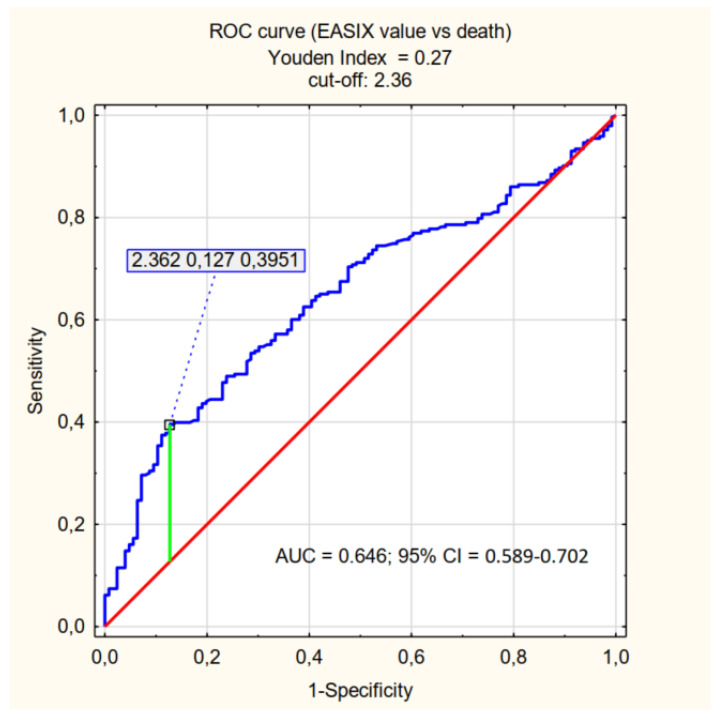
ROC curve (effect of EASIX value on death) = EASIX cut-off.

**Figure 2:**
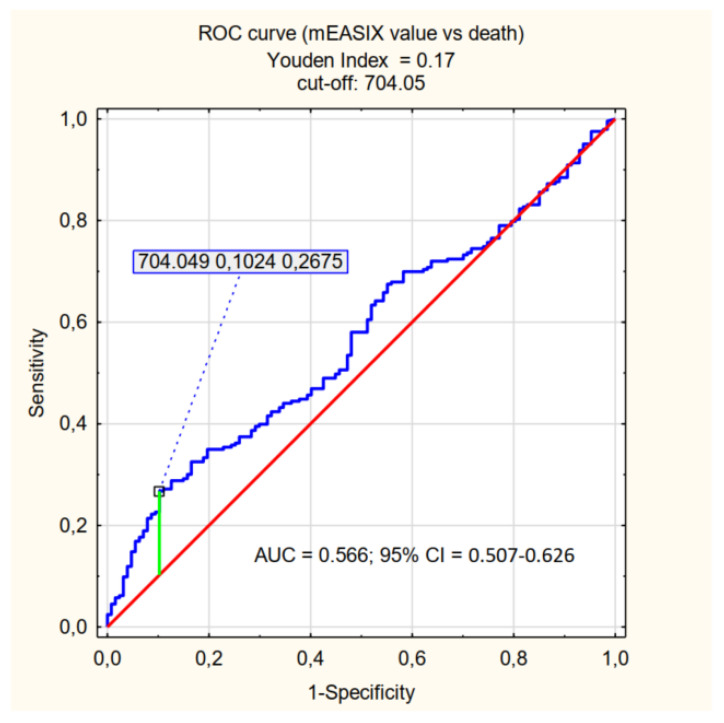
ROC curve (effect of mEASIX value on death) = mEASIX cut-off.

**Figure 3:**
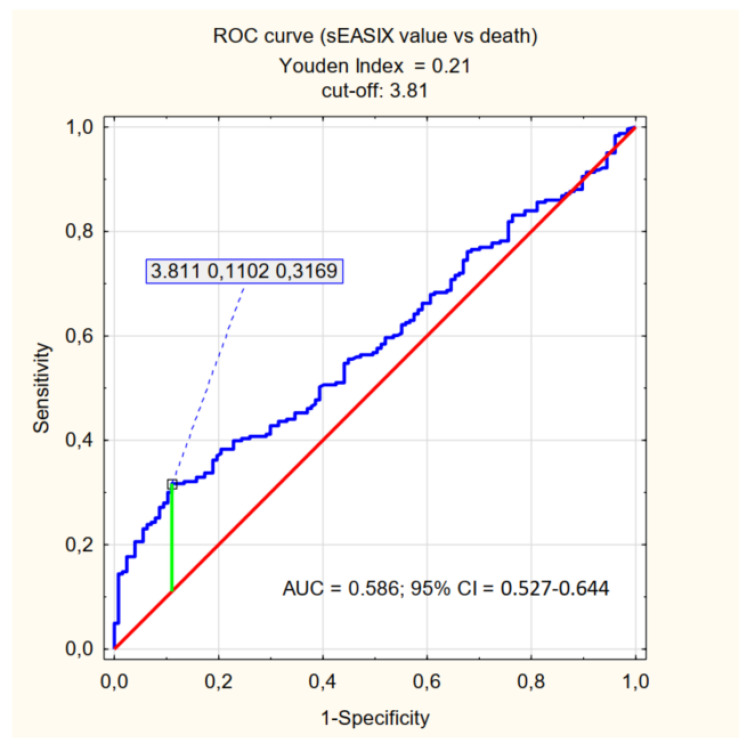
ROC curve (effect of sEASIX value on death) = sEASIX cut-off.

The cut-off values relevant for the prediction of mortality (determined by the Youden Index) were specified for the individual factors being assessed: log_2_-EASIX 2.36, log_2_-mEASIX 704.05, log_2_-sEASIX 3.81. Patients were divided into <cut-off and ≥cut-off groups, and the *p*-value was determined for each parameter–the characteristics of the assessed groups are presented in Tables S1–S3 (available in Supplementary Materials). The ability to explain the values of the factors satisfying the ≥cut-off criterion (referred to as high EASIX, high mEASIX, high sEASIX) is presented in Table 3 and Figure 1, Figure 2 and Figure 3.

In the group of patients with high EASIX, compared to those with low EASIX values, there were more male patients, more cases of circulatory system diseases and chronic renal failure, a greater frequency and shorter time of application of IMV, and significant differences in CRP, procalcitonin, ferritin, LDH, platelets, D-dimer, INR, AST and creatinine values. The high mEASIX and high sEASIX groups were characterised by a lower frequency and shorter time of application of HFNO, more frequent application of IMV and significant differences in CRP, procalcitonin, ferritin, LDH, lymphocytes, platelets, D-dimer, AST, ALT and creatinine values. Furthermore, higher INR values were observed in the high mEASIX group, which were not observed in the high sEASIX group. In turn, the high sEASIX group was also characterised by significant differences in the neutrophils count. In both modified coefficient groups (high mEASIX and high sEASIX), a shorter period between admission to the hospital and admission to the ICU was observed, while there were no differences with regard to the presence of comorbidities. For all coefficients being assessed, a significantly lower SpO_2_ value, shorter period of use of non-rebreather mask 10–15 L/min and a greater frequency of admission to the ICU were observed in the “high” groups. In turn, the patient’s age and haemoglobin concentration were not statistically significant in any of the groups, neither was diabetes, obesity and respiratory diseases. Males dominated the high EASIX and high mEASIX groups.

**Figure 4:**
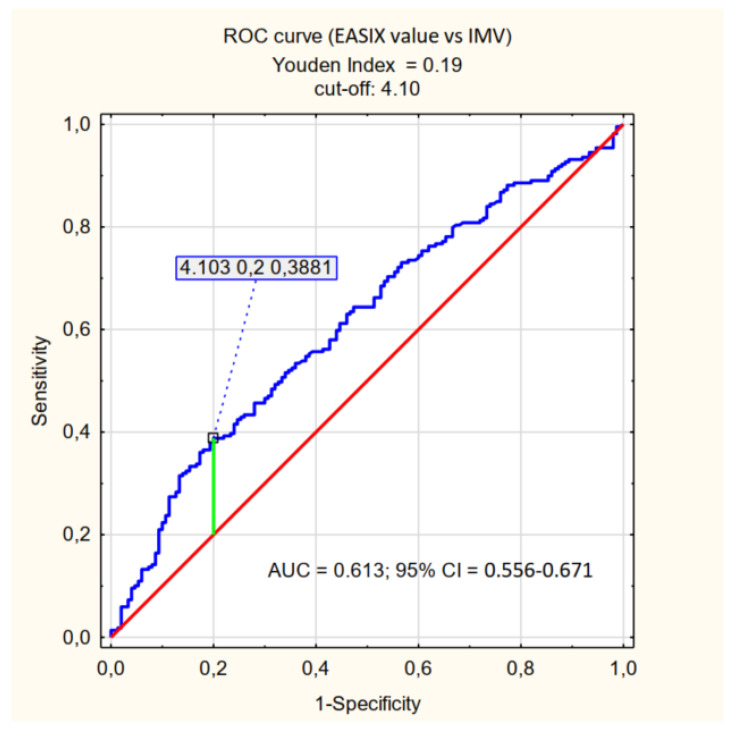
ROC curve (effect of EASIX value on IMV).

In the subsequent assessments of the course of hospitalisation, the values of the coefficients analysed were determined in the context of forecasting the use of particular methods of oxygen therapy. A significant correlation (*p* < 0.001) was only observed for IMV in the context of EASIX values with a cut-off point of 4.1 (Table 4, Figure 4). For the modified coefficients, the correlation was statistically insignificant.

## 5. Discussion

This study proves that all three indices, EASIX, mEASIX and sEASIX, are correlated with SARS-CoV-2 infection clinical outcomes, death and need for admission to the ICU. As they are not strong predictors in the group of severe COVID-19 patients, we do not recommend using them solely, but in addition to other more sensitive factors. Only for EASIX we observed a correlation with a risk of IMV treatment. We are the first to have analysed the above-mentioned indices in a selected group of patients with a severe course of infection. To our knowledge, this work is also the first publication to evaluate the usefulness of the modified coefficients (mEASIX and sEASIX) when it comes to assessing the risk of death in patients with COVID-19. The most important results of our study include: (i) the values of EASIX, mEASIX and sEASIX statistically significantly correlate with patient mortality, (ii) all three indices assessed for the population of patients with severe COVID-19 are characterised by low sensitivity (≤40%) with high specificity (approximately 90%) with regard to the assessment of the risk of death, (iii) high values of EASIX, mEASIX and sEASIX are more reliable in the assessment of the risk of death than survival, as evidenced by greater PPV compared to NPV, (iv) only EASIX is a predictor of the need to use IMV, but not HFNO and NIV; however, all three indices correlate with the need for admission to the ICU.

The high effectiveness of EASIX with regard to the stratification of COVID-19 patients was demonstrated in the study by Garcia et al. where, with the use of a retrospective analysis of more than 3000 patients, it was shown that the age-adjusted coefficient (aEASIX-COVID) has an even higher predictive value for mortality. It was also proved that, based on the values of both indices, EASIX and aEASIX, the chances of patient survival rather than death can be assessed more reliably, as illustrated by the significantly higher NPV (negative predictive value) compared to PPV (positive predictive value) [29]. In other words, with low values of EASIX and aEASIX, there is a greater probability of patient survival than death with high values. An equally significant (*p* < 0.001) correlation between EASIX and mortality in the course of COVID-19 was demonstrated in the study by Kalicinska et al., where two groups of patients were compared—a group of patients with haematological malignancies and patients with no haematological diseases. In addition, according to the study, EASIX is a strong predictor of the occurrence of acute kidney injury and hospitalisation in the ICU and, in the group of haematological patients, also of sepsis [28]. A similar correlation between EASIX and the need for the patient to be admitted to the ICU was determined in our study (*p* = 0.026).

According to the literature on the subject, there are further modifications of the EASIX index which, based on two studies—in groups of haematological patients with LBCL (large B-cell lymphoma) and B-ALL (B-cell acute lymphoblastic leukaemia)—proved to be significant predictors of complications after the application of CAR T-cell therapy (chimeric antigen receptor T-cells) in the form of CRS (cytokine release syndrome) and ICANS (immune effector cell-associated neurotoxicity syndrome). In their publication, Pennisi et al. used simplified EASIX (s-EASIX), where creatinine was excluded as a parameter that rarely showed a deviation in CRS patients, and modified-EASIX (m-EASIX), in which creatinine was replaced with CRP [31]. It turned out that all formulae applied prior to the treatment had a similar predictive value with regard to the occurrence of both complications, while only mEASIX allowed assessment of the effectiveness of the therapy. In the study by Greenbaum et al., EASIX was correlated with the concentration of ferritin (EASIX-F) and, in another case, with the concentration of ferritin and CRP (EASIX-FC) [32]. In this study, the modifications also had a positive effect and there was an increase in the predictive value in terms of the occurrence of complications due to the therapy.

In our study, we focused on the population of patients with severe COVID-19 and, also for this specific group of patients, we demonstrated predictive ability of EASIX, mEASIX and sEASIX in the context of mortality. Statistical significance was demonstrated for each parameter (*p* < 0.001). It should be noted, however, that for the established cut-offs (EASIX 2.36, mEASIX 704.05; sEASIX 3.81) the AUC are below 0.7, the sensitivity of the coefficients did not exceed 40% and they were characterised by high specificity (87%, 90%, and 89%, respectively). The values of PPV were also significantly greater than those of NPV, which should be understood as a greater probability of death with high values of the indices than the probability of survival with their low values. This is a somewhat surprising statistic, especially in the context of the publication by Garcia et al., in the case of which the obtained results are completely opposite (NPV higher than PPV) [29]. The cut-off value obtained in our study, which is significant for the prediction of death in the course of COVID-19 for EASIX (2.36), is greater than the one in the studies conducted by other researchers (Kalicińska et al. EASIX ≥ 1.6, Luft et al. EASIX ≥ 2.03) [27,28]. Such results may be influenced by the characteristics of the group of patients being assessed-patients in a critical condition, with a high probability of death (65.7%).

All three indices, EASIX, mEASIX and sEASIX, are not only predictors of mortality but also of the need for admission to the ICU (*p* = 0.026, *p* = 0.019, *p* = 0.001, respectively). Similar results with respect to EASIX were obtained in the research by Kalicińska et al. [28]. As part of our research, we also assessed the oxygen therapy methods applied, and only for EASIX was a statistically significant predictive value obtained for the use of IMV (cut off > 4.1, *p* < 0.001). Some of the patients being assessed who received IMV were hospitalised outside the ICU, which may have affected the statistical results. As for the mortality prediction, also in the context of that method of treatment, the sensitivity of that parameter was low (39%) with rather high specificity (80%). No similar correlation was observed for the other methods of oxygen therapy and for the modified indices.

Patients in the high EASIX (≥2.36), high mEASIX (≥704.05) and high sEASIX (≥3.81) groups compared to the “low” groups had significantly lower SpO_2_ values, shorter time of use of a non-rebreather mask 10–15 L/min, which resulted from disease progression and the need to intensify the treatment as well as the differences in the values of inflammatory parameters. The results of our analysis, in terms of laboratory parameters, are consistent with the results of studies by other researchers—they correspond to the pathology of the endothelium, which is associated with an intense inflammatory and thrombotic process in patients with severe COVID-19 [27,28,29,33,34,35]. Significantly, the modified indices (mEASIX and sEASIX) showed no differences when the “high” and “low” groups were compared with respect to comorbidities, and sEASIX also with respect to gender. The abovementioned results are very promising, although they need to be confirmed with further studies. They indicate the universality of the analysed coefficients and, above all, their independence from the gender of patients (sEASIX) and comorbidities (mEASIX, sEASIX).

The use of EASIX modification did not significantly increase the predictive capacity of the coefficient with respect to mortality (comparable results of the PPV, NPV, sensitivity and specificity analyses were achieved). Therefore, it seems that EASIX, mEASIX and sEASIX can be complementary used to assess patients with COVID-19 or other conditions with similar pathophysiology related to endothelial damage. Referring to the results of the study, EASIX in terms of patients death has an AUC that is closest to 0.7 (0.646) of all three indices and is the only one that relates to the necessity of an IMV, so it should be preferred to use, but even for this one his power is not sufficient. Due to the ease of implementation and the low cost of performing basic laboratory tests, those indices can be one of the tool for the initial assessment of patients admitted to the hospital, and thus facilitate and speed up the decision-making process with regard to the treatment of the patient, which may be crucial for prognosis. It is advisable to assess different variants of EASIX in a wider population of patients to find the most optimal one, that is to say the one with high specificity and sensitivity with regard to predicting mortality due to COVID-19.

This publication also has some limitations. The analysis is a retrospective one and it applies only to severely ill patients from the same institution; there is no control group and reference of the results to the group of patients with a mild course of the infection, which is the result of missing and impossible to obtain data. Therefore, the cut-off value achieved for EASIX is higher than the one reported in the previous publications and higher in relation to the expected values in the general population of patients infected with SARS-CoV-2. In the case of mEASIX and sEASIX, this is the first publication to assess those indices in the context of COVID-19, so the cut-off value cannot be compared to the previous results. Furthermore, this publication does not attempt to assess vaccination status and the course of hospitalisation, including methods of pharmacological treatment.

## 6. Conclusions

The EASIX, mEASIX, and sEASIX indices show a correlation with the risk of death and the need for admission to the ICU in patients with severe COVID-19. In the group of patients studied, in terms of the prediction of death, the high values of the three indices (EASIX ≥ 2.36, mEASIX ≥ 704.03, sEASIX ≥ 3.81) are characterised by low sensitivity (≤40%) and high specificity (approximately 90%). The highest AUC value (0.646) was achieved for EASIX. The research revealed that all three indices are not optimal diagnostic tools in to severely ill patients, but they might be used additionally to other more sensitive predictors. High values of EASIX, mEASIX and sEASIX are more reliable in assessing the risk of death than the probability of survival and, additionally, EASIX is a predictor of the need to apply IMV (invasive mechanical ventilation).

## Figures and Tables

**Table 1:** Characteristics of the group—the variables relate to the time of admission to the hospital; quantitative parameters and variables are presented as mean values (standard deviation-SD), median [interquartile range-IQR]; qualitative variables are presented as frequency/number. Standards for laboratory parameters are given in brackets next to each one.

		Clinical Outcome		
Variable	No. of Patients, *n* = 370	Non-Survivors, *n* = 243	Survivors, *n* = 127	*p*-Value
Male	242 (65.4%)	155 (63.8%)	87 (68.5%)	0.365
Age	66.9 (12.9), 68 [60.8–74.3]	69.7 (11.4), 70 [63–77]	61.6 (14), 65 [52–70]	**<0.001**
Death	243 (65.7%)			
Concomitant diseases				
Cardiovascular diseases	251 (67.8%)	176 (72.4%)	75 (59.1%)	**0.009**
Hypertension	231 (62.4%)	161 (66.3%)	70 (55.1%)	**0.036**
Atrial fibrillation	47 (12.7%)	38 (15.6%)	9 (7.1%)	**0.019**
Ischemic heart disease	86 (23.2%)	70 (28.8%)	16 (12.6%)	**<0.001**
Pulmonary diseases	38 (9.7%)	26 (10.7%)	10 (7.9%)	0.384
Asthma	19 (5.1%)	13 (5.3%)	6 (4.7%)	0.796
COPD	11 (3.0%)	8 (3.3%)	3 (2.4%)	0.617
Other	8 (2.2%)	7 (2.9%)	1 (0.8%)	0.189
Diabetes	134 (36.2%)	97 (39.9%)	37 (29.1%)	0.103
Malignant neoplasm	57 (15.4%)	44 (18.1%)	13 (10.2%)	**0.046**
Chronic kidney disease	33 (8.9%)	30 (12.3%)	3 (2.4%)	**0.001**
Obesity	105 (28.4%)	68 (28.0%)	37 (29.1%)	0.816
SpO_2_ without oxygen supply	83.4 (12.1), 86 [80–92]	82.4 (13.6), 86.5 [78–92]	85.4 (7.92), 86 [82–90]	0.442
Severity (according to WHO scale)				0.171
1	5 (1.4%)	3 (1.2%)	2 (1.6%)	
2	103 (27.8%)	69 (28.4%)	34 (26.8%)	
3	222 (60.0%)	136 (56.0%)	86 (67.7%)	
4	40 (10.8%)	35 (14.4%)	5 (3.9%)	
Duration of hospitalisation, days	19.3 (15.2), 16 [10–24]	16.1 (12.6), 14 [8–21]	25.5 (17.7), 21 [13–31.8]	**<0.001**
Duration of symptoms before hospital admission, days	7.1 (4.4), 7 [4–9]	6.7 (4.5), 6 [4–8]	7.8 (4.1), 7 [5–10]	**0.026**
Deterioration, days (from disease onset)	8.5 (5.8), 7 [5–10]	8.4 (6.6), 7 [5–10]	8.8 (4.2), 8 [6–11]	0.564
ICU admission	216 (58.4%)	182 (74.9%)	34 (26.8%)	**<0.001**
Non-rebreather mask 10–15 L/min	352 (95.1%)	229 (94.2%)	123 (96.9%)	0.268
Non-rebreather mask 10–15 L/min duration, days	2.6 (3.3), 2 [1–3]	2.6 (3.6), 2 [1–3]	2.6 (2.6), 2 [1–3]	0.925
HFNO	253 (68.4%)	147 (60.5%)	106 (83.5%)	**<0.001**
HFNO duration, days	5.6 (5.0), 4 [2–8]	3.6 (3.2), 2 [1–5]	8.3 (5.8), 7 [4–11]	**<0.001**
NIV	69 (18.6%)	60 (24.7%)	9 (7.1%)	**<0.001**
NIV duration, days	4.9 (4.8), 3 [1–6.5]	4.8 (4.9), 3 [1.3–6]	5.1 (5.4), 3 [1–7]	0.829
IMV	220 (59.5%)	186 (76.5%)	34 (26.8%)	**<0.001**
IMV duration, days	13.8 (12.9), 11 [6–17]	12.3 (11.0), 10 [5.5–17]	22.6 (19.3), 17 [11.3–26.3]	**0.001**
Lab test results				
CRP (>6 mg/L)	140.30 (99.23), 123.40 [62–199.2]	142.30 (100.60), 125.00 [63.59–198.30]	136.40 (97.20), 117.6 [59.90–201.10]	0.478
Procalcitonin (>0.05 ng/mL)	1.74 (4.57), 0.26 [0.1–0.86]	2.12 (5.14), 0.35 [0.13–1.14]	1.08 (3.31), 0.15 [0.08–0.39]	0.110
Ferritin (>274.66 ng/mL	1846.93 (2210.77), 1527.24 [593.86–2325.59]	1910.18 (2228.75), 1304.80 [632.49–2489.59]	1763.91 (2212.21), 1138.11 [566.72–2308.85]	0.699
LDH (>220 U/L)	575 (315), 530 [397–671]	602 (368), 550 [388–734]	524 (161), 504 [411–608]	**0.026**
White blood count (4–10 G/L)	9.00 (9.51), 7.56 [5.46–10.49]	9.47 (11.4), 7.99 [5.47–11.02]	8.09 (3.83), 7.39 [5.43–9.77]	0.180
Neutrophils (1.8–7 G/L)	7.10 (4.1), 6.3 [4.1–8.8]	7.22 (4.29), 6.5 [4.1–9.5]	6.75 (3.73), 6.00 [4.28–8.03]	0.362
Lymphocytes (1–4 G/L)	1.00 (0.7), 0.8 [0.6–1.2]	1.00 (0.8), 0.8 [0.6–1.2]	0.90 (0.4), 0.8 [0.6–1.1]	0.188
Haemoglobin (13.5–18.0 g/dL)	13.20 (2.21), 13.4 [12.1–14.7]	12.90 (2.39), 13.1 [11.8–14.5]	13.70 (1.71), 14.10 [12.7–14.9]	0.860
Thrombocytes (150–420 G/L)	222 (106), 197 [152–265]	216 (109), 192 [145–260]	233 (99.9), 206 [167–277]	0.130
D-dimer (>500 ng/mL)	4073.30 (10,041.6), 1409.5 [876.5–2676.75]	5163.78 (12,060), 1797 [976–3327]	2060.79 (3681.20), 1145.00 [728.00–2104.00]	**0.018**
INR (0.8–1.2)	1.17 (0.37), 1.09 [1.02–1.19]	1.20 (0.41), 1.11 [1.03–1.23]	1.12 (0.29), 1.06 [1.00–1.14]	0.073
AST (>34 U/L)	69.34 (57.95), 54.70 [39.00–80.23]	71.07 (61.80), 53 [38.85–82.90]	66.24 (50.73), 57.00 [39.30–73.00]	0.479
ALT (>55 U/L)	52.83 (54.03), 39.35 [25.85–64.40]	51.02 (60.1), 37 [22.85–63.45]	56.27 (40.37), 42.80 [29.20–68.60]	0.386
Creatinine (>1.15 mg/dL)	1.43 (1.57), 1.03 [0.81–1.44]	1.58 (1.78), 1.13 [0.83–1.66]	1.14 (1.03), 0.92 [0.78–1.11]	**0.016**
EASIX	5.39 (11.04), 2.73 [1.68–4.82]	6.66 (13.29), 3.11 [1.89–5.87]	2.94 (2.92), 2.05 [1.59–3.18]	**<0.001**
mEASIX	497.49 (783.11), 303.36 [130.07–598.69]	571.22 (924.56), 315.37 [130.23–740.82]	356.42 (358.55), 264.14 [125.57–468.00]	**<0.001**
sEASIX	3.40 (4.76), 2.49 [1.81–3.79]	3.86 (5.77), 2.60 [1.88–4.25]	2.52 (1.18), 2.36 [1.74–3.23]	**<0.001**

* COPD—Chronic Obstructive Pulmonary Disease; ICU—Intensive Care Unit, HFNO—High Flow Nasal Oxygenation; NIV—Non Invasive Ventilation; IMV—Invasive Mechanical Ventilation; CRP—C-Reactive Protein; LDH—Lactate Dehydrogenase activity; INR—International Normalised Ratio; AST—Aspartate Aminotransferase activity; ALT—Alanine Aminotransferase activity; EASIX—Endothelial Activation and Stress Index, mEASIX—modified-EASIX, sEASIX—simplified-EASIX.

**Table 2:** AUC, cut-off, sensitivity and specificity for predicting mortality in a patient with COVID-19 according to parameters assessed on admission with *p* < 0.05 (determined by Youden Index).

	*p*-Value	Cut-Off	AUC	95% CI	Sensitivity	Specificity
Variable						
Age	<0.001	≥72	0.662	0.603–0.720	0.44	0.80
Duration of symptoms before hospital admission, days	0.026	<7	0.581	0.520–0.642	0.72	0.40
Lab test parameters						
LDH	0.026	≥656	0.540	0.482–0.599	0.34	0.86
D-dimer	0.018	≥1714	0.636	0.576–0.696	0.53	0.68
Creatinine	0.016	≥1.23	0.642	0.585–0.698	0.44	0.85

**Table 3:** Sensitivity, specificity, PPV (positive predictive value), NPV (negative predictive value), AUC for predicting mortality in patients with COVID-19 according to EASIX, mEASIX and sEASIX cut-offs.

	AUC	95% CI	Sensitivity	Specificity	PPV	NPV
High EASIX	0.646	0.589–0.702	0.40	0.87	0.86	0.43
High mEASIX	0.566	0.507–0.626	0.27	0.90	0.83	0.38
High sEASIX	0.586	0.527–0.644	0.32	0.89	0.83	0.41

**Table 4:** Correlation of EASIX values (cut-off, AUC, sensitivity, specificity) with regard to the need to apply particular methods of oxygen therapy.

	EASIX					
Effect of:	*p*	Cut-Off	AUC	95% CI	Sensitivity	Specificity
HFNO	0.622					
NIV	0.271					
IMV	**<0.001**	4.10	0.613	0.556–0.671	0.39	0.80

## Data Availability

Not applicable.

## Supplementary Materials

The following supporting information can be downloaded at: https://www.mdpi.com/article/10.3390/jpm12071022/s1, Table S1: Correlation of EASIX with variables being assessed and characteristics of patients with high EASIX (≥2.36) and low EASIX (<2.36); Table S2: Correlation of mEASIX with the variables being assessed and characteristics of patients with high mEASIX (≥704.05) and low mEASIX (<704.05); Table S3: Correlation of sEASIX with variables being assessed and characteristics of patients with high sEASIX (≥3.81) and low sEASIX (<3.81).
