# Supplementary material for: EASIX, Modified EASIX and Simplified EASIX as an Early Predictor for Intensive Care Unit Admission and Mortality in Severe COVID-19 Patients
Source: J Pers Med. 2022 Jun 21;12(7):1022. doi: 10.3390/jpm12071022 (PMC9321320; doi:10.3390/jpm12071022)
Supplement: Supplementary file 1 [file jpm-12-01022-s001.zip › jpm-1741412-supplementary.pdf]

**Table S1.** Correlation of EASIX with variables being assessed and characteristics of patients with high EASIX (≥2.36) and low EASIX (<2.36). Parameters and quantitative variables are presented as median [interquartile range - IQR]; qualitative variables are presented as frequency/number.

| | **EASIX *p*-value** | **High EASIX** | **Low EASIX** | ***p*-value** |
|---|---|---|---|---|
| Variable | | *n* = 111 | *n* = 258 | |
| Male | **<0.001** | 82 (73.9%) | 159 (66.0%) | **0.023** |
| Age | 0.125 | 68 [61–76] | 68 [60–74] | 0.639 |
| Death | **<0.001** | 95 (85.6%) | 148 (57.4%) | **<0.001** |
| Concomitant diseases | | | | |
| Cardiovascular diseases | **<0.001** | 87 (78.4%) | 163 (63.2%) | **0.004** |
| Hypertension | **<0.001** | 82 (73.9%) | 148 (%) | **0.003** |
| Atrial fibrilation | 0.110 | 17 (15.3%) | **30 (11.6%)** | 0.330 |
| Ischemic heart disease | 0.050 | 34 (30.6%) | **52 (20.2%)** | **0.029** |
| Pulmonary diseases | 0.345 | 8 (7.2%) | 28 (10.9%) | 0.279 |
| Asthma | 0.894 | 5 (4.5%) | 14 (5.4%) | 0.713 |
| COPD | 0.951 | 3 (2.7%) | 8 (3.1%) | 0.837 |
| Other | 0.424 | 2 (1.8%) | 6 (2.3%) | 0.751 |
| Diabetes | 0.223 | 46 (41.4%) | 88 (34.1%) | 0.179 |
| Malignant neoplasm | **0.033** | 13 (11.7%) | 44 (17.1%) | 0.193 |
| Chronic kidney disease | **<0.001** | 25 (22.3%) | 8 (3.1%) | **<0.001** |
| Obesity | 0.925 | 31 (27.9%) | 74 (28.7%) | 0.883 |
| $SpO_2$ without oxygen supply | **0.014** | 83 [70–90] | 87 [82–92] | **<0.001** |
| Severity (according to WHO scale) | **0.027** | | | 0.076 |
| 1 | | 1 (0.9%) | 4 (1.6%) | |
| 2 | | 27 (24.3%) | 76 (29.5%) | |
| 3 | | 64 (57.7%) | 157 (60.8%) | |
| 4 | | 19 (17.1%) | 21 (8.1%) | |
| Duration of hospitalisation, days | **0.019** | 12 [6–20] | 18 [12–25] | **<0.001** |
| Duration of symptoms before hospital admission, days | 0.549 | 6 [4–8] | 7 [5–9] | 0.181 |
| Deterioration, days (from disease onset) | **<0.001** | 7 [4–9] | 8 [5–12] | **0.002** |
| ICU admission | **0.026** | 80 (72.1%) | 135 (52.3%) | **0.001** |
| ICU admission, days (from disease onset) | **0.032** | 9 [7–16] | 12 [8–17] | 0.102 |
| ICU admission, days (from hospitalisation onset) | **0.042** | 3 [1–7] | 4 [2–8] | 0.062 |
| ICU duration, days | **<0.001** | 9 [6–17] | 13 [8–20] | **0.009** |
| Non-rebreather mask 10-15l/min | 0.764 | 104 (93.7%) | 247 (95.7%) | 0.403 |
| Non-rebreather mask 10-15l/min duration, days | **<0.001** | 1 [0–2] | 2 [1–3] | **<0.001** |
| HFNO | 0.622 | 72 (64.9%) | 181 (70.2%) | 0.315 |
| HFNO duration, days | **<0.001** | 2 [1–6] | 5 [2–9] | **<0.001** |
| NIV | 0.271 | 24 (21.8%) | 45 (17.4%) | 0.325 |
| NIV duration, days | 0.835 | 3 [1–5] | 4 [1–7] | 0.604 |
| IMV | **<0.001** | 82 (74.6%) | 137 (53.1%) | **<0.001** |
| IMV duration, days | **0.008** | 8 [5–16] | 12 [7–19] | **0.006** |

| | | | | |
|---|---|---|---|---|
| Lab test results | | | | |
| CRP | **0.001** | 143.2 [78.5–214.7] | 112.3 [55.7–188.5] | **0.012** |
| Procalcitonin | **<0.001** | 0.722 [0.283–2.89] | 0.157 [0.084–0.474] | **<0.001** |
| Ferritin | **<0.001** | 2137.8 [985.3–3608.3] | 1037 [526.7–1611.6] | **<0.001** |
| LDH | **<0.001** | 685 [530–897] | 471 [376–592] | **<0.001** |
| White blood count | **0.003** | 7.06 [5.13–9.55] | 8.04 [5.81–10.78] | 0.079 |
| Neutrophils | **0.011** | 5.6 [4.05–8] | 6.6 [4.2–8.9] | 0.147 |
| Lymphocytes | **0.021** | 0.8 [0.6–1.1] | 0.9 [0.6–1.2] | 0.067 |
| Haemoglobin | 0.326 | 13.3 [11.6–14.7] | 13.4 [12.3–14.7] | 0.608 |
| Thrombocytes | **<0.001** | 159 [128–196] | 223 [170–297] | **<0.001** |
| D-dimer | **0.030** | 2017 [1037–3326] | 1264 [822–2424] | **<0.001** |
| INR | **0.042** | 1.12 [1.04–1.3] | 1.08 [1.01–1.16] | **0.001** |
| AST | **<0.001** | 69.4 [46.6–105] | 50 [36.2–67.8] | **<0.001** |
| ALT | 0.146 | 42 [25.9–69] | 38 [25–63.3] | 0.313 |
| Creatinine | **<0.001** | 1.71 [1.28–2.4] | 0.91 [0.76–1.13] | **<0.001** |

**Table S2.** Correlation of mEASIX with the variables being assessed and characteristics of patients with high mEASIX (≥704.05) and low mEASIX (<704.05). Parameters and quantitative variables are presented as median [interquartile range— IQR]; qualitative variables are presented as frequency/number.

| | **mEASIX *p*-value** | **High mEASIX** | **Low mEASIX** | ***p*-value** |
|---|---|---|---|---|
| Variable | | *n* = 124 | *n* = 246 | |
| Male | **<0.001** | 93 (75.0%) | 149 (60.6%) | **0.006** |
| Age | **0.032** | 67 [60–74] | 68 [61–74.5] | 0.682 |
| Death | **<0.001** | 91 (73.4%) | 152 (61.8%) | **0.027** |
| Concomitant diseases | | | | |
| Cardiovascular diseases | 0.334 | 89 (71.8%) | 162 (65.9%) | 0.569 |
| Hypertension | 0.672 | 79 (63.7%) | 152 (61.8%) | 0.719 |
| Atrial fibrilation | 0.484 | 21 (16.9%) | 26 (10.6%) | 0.083 |
| Ischemic heart disease | 0.723 | 32 (25.8%) | 54 (22.0%) | **0.407** |
| Pulmonary diseases | 0.608 | 11 (8.9%) | 25 (10.2%) | 0.692 |
| Asthma | 0.388 | 9 (7.3%) | 10 (4.1%) | 0.189 |
| COPD | 0.875 | 3 (2.4%) | 8 (3.3%) | 0.656 |
| Other | 0.196 | 1 (0.8%) | 7 (2.8%) | 0.203 |
| Diabetes | 0.474 | 46 (37.1%) | 88 (35.8%) | 0.802 |
| Malignant neoplasm | **0.026** | 16 (12.9%) | 41 (16.7%) | 0.344 |
| Chronic kidney disease | 0.793 | 8 (6.5%) | 24 (9.8%) | 0.286 |
| Obesity | 0.843 | 33 (26.6%) | 72 (29.3%) | 0.593 |
| $SpO_2$ without oxygen supply | **<0.001** | 80 [68–88] | 87 [81–92] | **<0.001** |
| Severity (according to WHO scale) | | | | |
| 1 | 0.296 | 0 (0.0%) | 5 (2.0%) | **<0.001** |
| 2 | | 17 (13.7%) | 86 (35.0%) | |
| 3 | | 87 (70.2%) | 135 (54.9%) | |
| 4 | | 20 (16.1%) | 20 (8.1%) | |
| Duration of hospitalisation, days | 0.226 | 12 [5–20] | 17 [11–25] | **<0.001** |
| Duration of symptoms before hospital admission, days | **0.025** | 7 [5–8] | 7 [4–9] | 0.617 |
| Deterioration, days (from disease onset) | 0.064 | 7 [5–9] | 8 [5–10] | 0.199 |
| ICU admission | **0.019** | 84 (67.7%) | 132 (53.7%) | **0.017** |

| | | | | |
|---|---|---|---|---|
| ICU admission, days (from disease onset) | 0.098 | 9 [6.5–15] | 12 [7–17] | 0.050 |
| ICU admission, days (from hospitalisation onset) | 0.241 | 2 [0–4] | 5 [2–8] | **<0.001** |
| ICU duration, days | 0.134 | 11 [5–18] | 11.5 [6–18] | 0.314 |
| Non-rebreather mask 10-15l/min | 0.127 | 114 (91.9%) | 238 (96.8%) | **0.042** |
| Non-rebreather mask 10-15l/min duration, days | 0.321 | 1 [0–2] | 2 [1–3] | **<0.001** |
| HFNO | 0.959 | 79 (63.7%) | 174 (70.7%) | 0.170 |
| HFNO duration, days | 0.126 | 2 [1–5] | 5 [2–9] | **<0.001** |
| NIV | 0.133 | 27 (21.8%) | 42 (17.1%) | 0.273 |
| NIV duration, days | 0.057 | 3 [2–5] | 4 [1–6] | 0.741 |
| IMV | 0.138 | 87 (70.2%) | 133 (54.1%) | **0.003** |
| IMV duration, days | 0.178 | 10 [4–16] | 11 [6–18] | 0.062 |
| | | Lab test results | | |
| CRP | **<0.001** | 233.3 [180.2–296.1] | 96.4 [51.6–155.7] | **<0.001** |
| Procalcitonin | **<0.001** | 0.91 [0.25–1.69] | 0.16 [0.07–0.49] | **<0.001** |
| Ferritin | **<0.001** | 2321 [979–4474] | 1086 [546–1750] | **<0.001** |
| LDH | **<0.001** | 777 [588–954] | 476 [374–602] | **<0.001** |
| White blood count | 0.245 | 8.18 [5.49–11.08] | 7.55 [5.51–10.34] | 0.671 |
| Neutrophils | **0.003** | 6.7 [4.6–10.0] | 6 [3.9–8.6] | 0.162 |
| Lymphocytes | **<0.001** | 0.7 [0.5–0.9] | 0.9 [0.6–1.2] | **0.004** |
| Haemoglobin | 0.711 | 13.3 [10.9–14.6] | 13.4 [12.2–14.7] | 0.216 |
| Thrombocytes | **<0.001** | 153 [123–204] | 211 [163–281] | **<0.001** |
| D-dimer | **<0.001** | 2180 [1173–3950] | 1258 [797–2426] | **<0.001** |
| INR | **<0.001** | 1.14 [1.05–1.22] | 1.08 [1.0–1.1] | **0.002** |
| AST | **<0.001** | 74 [52–106] | 50 [36–69] | **<0.001** |
| ALT | **<0.001** | 46 [31–82] | 37 [25–61] | **0.005** |
| Creatinine | **<0.001** | 1.36 [0.98–1.79] | 0.99 [0.79–1.29] | **<0.001** |

**Table S3.** Correlation of sEASIX with variables being assessed and characteristics of patients with high sEASIX (≥3.81) and low sEASIX (<3.81). Parameters and quantitative variables are presented as median [interquartile range—IQR]; qualitative variables are presented as frequency/number.

| | **sEASIX *p*-value** | **High sEASIX** | **Low sEASIX** | ***p*-value** |
|---|---|---|---|---|
| Variable | | *n* = 91 | *n* = 279 | |
| Male | 0.052 | 60 (66.7%) | 182 (65.2%) | 0.903 |
| Age | 0.087 | 68 [60–74] | 68 [61–75] | 0.582 |
| Death | **<0.001** | 77 (84.6%) | 166 (59.5%) | **<0.001** |
| | | Concomitant diseases | | |
| Cardiovascular diseases | **0.027** | 69 (75.8%) | 182 (65.2%) | 0.060 |
| Hypertension | 0.319 | 65 (72.2%) | 166 (59.5%) | 0.051 |
| Atrial fibrilation | 0.411 | 14 (15.6%) | 33 (11.8%) | 0.376 |
| Ischemic heart disease | 0.644 | 23 (25.6%) | 63 (22.6%) | 0.597 |
| Pulmonary diseases | 0.782 | 10 (11.0%) | 26 (9.3%) | 0.641 |
| Asthma | 0.358 | 7 (7.8%) | 12 (4.3%) | 0.203 |
| COPD | 0.963 | 3 (3.3%) | 8 (2.9%) | 0.834 |
| Other | 0.948 | 2 (2.2%) | 6 (2.2%) | 0.979 |
| Diabetes | 0.658 | 36 (39.6%) | 98 (35.1%) | 0.614 |
| Malignant neoplasm | **0.046** | 15 (16.5%) | 42 (15.1%) | 0.743 |
| Chronic kidney disease | 0.420 | 11 (12.1%) | 22 (7.9%) | 0.222 |
| Obesity | 0.799 | 22 (24.2%) | 83 (29.8%) | 0.306 |
| $SpO_2$ without oxygen supply | **0.001** | 84 [72–90] | 87 [80–92] | **0.015** |

| | | | | |
|---|---|---|---|---|
| Severity (according to WHO scale)<br>1<br>2<br>3<br>4 | 0.095 | <br><br>1 (1.1%)<br>22 (24.4%)<br>51 (56.6%)<br>17 (18.9%) | <br><br>4 (1.4%)<br>81 (29.1%)<br>171 (61.3%)<br>23 (8.2%) | **0.049** |
| Duration of hospitalisation, days | 0.160 | 13.5 [7–21] | 17 [11–25] | **0.003** |
| Duration of symptoms before hospital admission, days | 0.949 | 7 [5–8] | 7 [4–9] | 0.860 |
| Deterioration, days (from disease onset) | 0.089 | 7 [5–9] | 8 [5–11] | 0.306 |
| ICU admission | **0.001** | 66 (73.3%) | 150 (53.8%) | **0.004** |
| ICU admission, days (from disease onset) | 0.078 | 10 [7–15.5] | 12 [7–17] | 0.301 |
| ICU admission, days (from hospitalisation onset) | 0.154 | 3 [0–6] | 4 [2–8] | **0.033** |
| ICU duration, days | **0.035** | 11 [6–17.5] | 11 [6–18] | 0.621 |
| Non-rebreather mask 10-15l/min | 0.300 | 84 (92.3%) | 268 (96.1%) | 0.149 |
| Non-rebreather mask 10-15l/min duration, days | 0.187 | 1 [0–3] | 2 [1–3] | **0.001** |
| HFNO | 0.825 | 53 (58.2%) | 200 (71.7%) | **0.017** |
| HFNO duration, days | 0.161 | 2 [1–4] | 5 [2–9] | **<0.001** |
| NIV | 0.404 | 17 (18.9%) | 52 (18.6%) | 0.958 |
| NIV duration, days | **0.003** | 3 [1–7] | 3 [1–6] | 0.871 |
| IMV | 0.134 | 68 (75.6%) | 152 (54.5%) | **<0.001** |
| IMV duration, days | 0.056 | 10 [6–17] | 11 [6–17] | 0.490 |
| | | Lab test results | | |
| CRP | **0.011** | 150.4 [86.3–220.1] | 113.9 [57.7–186.8] | **0.018** |
| Procalcitonin | **<0.001** | 0.49 [0.19–1.21] | 0.18 [0.09–0.66] | **<0.001** |
| Ferritin | **<0.001** | 2116 [740–3340] | 1146 [566–1672] | **0.004** |
| LDH | **<0.001** | 772 [619–935] | 464 [374–588] | **<0.001** |
| White blood count | **<0.001** | 7.02 [4.66–9.04] | 8.21 [5.81–10.97] | **0.002** |
| Neutrophils | **<0.001** | 5.4 [3.7–7.9] | 6.7 [4.2–9.4] | **0.010** |
| Lymphocytes | **<0.001** | 0.8 [0.5–1.0] | 0.9 [0.6–1.2] | **0.009** |
| Haemoglobin | 0.089 | 13.2 [11.8–14.7] | 13.5 [12.2–14.7] | 0.512 |
| Thrombocytes | **<0.001** | 148 [116–183] | 226 [172–297] | **<0.001** |
| D-dimer | 0.286 | 1798 [1046–3442] | 1366 [818–2644] | **0.034** |
| INR | 0.848 | 1.12 [1.03–1.24] | 1.08 [1.02–1.19] | 0.122 |
| AST | **<0.001** | 74 [50–106] | 50 [36–68] | **<0.001** |
| ALT | **<0.001** | 43 [30–76] | 37 [25–61] | **0.032** |
| Creatinine | **<0.001** | 1.27 [0.87–1.79] | 1 [0.8–1.32] | **0.002** |
